# P12 A periorbital and upper back rash on a female patient of West African descent: a case of NXP2 dermatomyositis initially misdiagnosed as an allergic reaction

**DOI:** 10.1093/rap/rkad070.033

**Published:** 2023-09-27

**Authors:** Jacob Williams, Ryan Malcolm Hum, Pauline Ho

**Affiliations:** The Kellgren Centre for Rheumatology, Manchester University NHS Foundation Trust, Manchester, United Kingdom; The Kellgren Centre for Rheumatology, Manchester University NHS Foundation Trust, Manchester, United Kingdom; Centre for Musculoskeletal Research, The University of Manchester, Manchester, United Kingdom; The Kellgren Centre for Rheumatology, Manchester University NHS Foundation Trust, Manchester, United Kingdom; Centre for Musculoskeletal Research, The University of Manchester, Manchester, United Kingdom

## Abstract

**Introduction:**

Idiopathic inflammatory myopathies (IIM) are a group of conditions characterised by immune-driven muscular inflammation, often in the presence of specific autoantibodies. NXP2 dermatomyositis is a subtype of IIM characterised by antibodies to nuclear matrix protein 2. NXP2 dermatomyositis is associated with calcinosis, especially in younger patients and with malignancy, especially in older patients. We report a case of NXP2 dermatomyositis in a female patient of West African descent presenting with a periorbital and upper back rash which was initially misdiagnosed as an allergic reaction. Images of darker skin are underrepresented in medical education around dermatomyositis.

**Case description:**

A 49-year-old female office worker presented to A&E with a two-week history of arthralgia and fatigue. Over the past two days, she had also noticed the development of periorbital swelling and a rash on her eyelids and her upper back. On examination in A&E, the rash was considered minor and it was noted that she had difficulty taking off her coat due to pain in her arms. She was discharged home with a working diagnosis of an allergic reaction, with advice to see her GP if symptoms persisted.

She was reviewed by her GP, who suspected a vasculitic rash. She was found to have a significantly raised ANCA-MPO antibody titre and was referred to the acute medical take where she was seen urgently by rheumatology on-call. On assessment, she was noted to have pain and morning stiffness in her hands and wrists, with swollen DIPJ, MCPJ and wrists on examination. On assessment by a rheumatologist aware of the skin manifestations of dermatomyositis, she was also noted to have a heliotrope rash, shawl sign, periungual erythema, and a V sign rash and was suspected to have dermatomyositis. Her CK was raised at 3000, and she was found to have NXP2 autoantibodies which confirmed the diagnosis.

She was started on hydroxychloroquine whilst an MR scan of her arms and legs was organised, which revealed evidence of myositis in the proximal muscles. Following MR scanning, she was started on high dose corticosteroids. Azathioprine was subsequently initiated as a steroid sparing agent, but was not tolerated due to nausea and so she was switched to subcutaneous methotrexate. Her CK has now normalised, and her skin lesions are healing.

**Discussion:**

One of the key issues highlighted by this case is the lack of awareness of rashes associated with dermatomyositis generally, and especially in diverse skin types. We suspect this is because of a lack of education and exposure to skin rashes in diverse skin types, especially in highly melanated skin types. On her initial assessment in A&E, her rash was misdiagnosed as an allergic reaction. We suspect a lack of familiarity with skin rashes in darker skin types contributed to this initial misdiagnosis. However, we believe it was clear that the patient’s rash and history on initial presentation were not in keeping with urticaria, or an allergic skin rash. However, a further point to consider is that dermatomyositis is rare, and so those working in primary care are less familiar with its manifestations.

Medical resources have historically had a tendency to use images of people with lighter skin tones to demonstrate skin diseases. Over the last few years, several textbooks and online resources have become available specifically showing skin conditions in different skin tones, for example, the Mind the Gap handbook. These resources are generally excellent; however, they tend to focus on common skin disorders. In addition, images of dermatomyositis in medical textbooks are predominantly of white skin. If medical education resources were to include examples of dermatomyositis in a variety of skin tones it is possible that diagnostic confidence and proficiency would improve, and racial disparities would be reduced. This is especially important in a multicultural society like the United Kingdom.

**Key learning points:**

Lack of awareness of skin rashes in diverse skin types contributes to misdiagnosis and worse outcomes for patients with ethnic minority backgrounds, including those with dermatomyositis.

Increasing awareness and education surrounding the skin manifestations in diverse skin types is a key priority for medical education, including in rheumatology.

New initiatives aimed at improving our understanding of skin manifestations in diverse skin types are underway.

